# Myocardial dissection complicating left sinus of Valsalva aneurysm in silent takayasu arteritis

**DOI:** 10.1186/s12872-021-02271-4

**Published:** 2021-09-26

**Authors:** Astri Astuti, Achmad Hafiedz Azis Kartamihardja, Muhammad Adniel Ilhamy, Muhammad Dinnar Fahlavi, Nuraini Yasmin Kusumawardhani, Melawati Hasan, Laniyati Hamijoyo

**Affiliations:** 1 Department of Cardiology and Vascular Medicine, Hasan Sadikin General Hospital, Universitas Padjadjaran, Jl. Pasteur no.38, Bandung, West Java Indonesia; 2 Division of Rheumatology, Department of Internal Medicine, Hasan Sadikin General Hospital, Universitas Padjadjaran, Bandung, Indonesia

**Keywords:** Myocardial dissection, Left sinus of Valsalva aneurysm, Takayasu arteritis

## Abstract

**Background:**

Myocardial dissection (MD) in a left sinus of Valsalva aneurysm (LSVA) is a rare condition that may lead to a fatal complication. Determining the MD etiology is challenging because of various possibilities ranging from congenital to acquired diseases. Here, we discuss an approach for determining the etiology of MD complicating LSVA in Takayasu arteritis (TA) and its treatment.

**Case presentation:**

A 41-year-old man presented with dyspnea on heavy activities and a history of consciousness loss at the age of 24 years. He was diagnosed with dilated cardiomyopathy and MD complicating LSVA in TA based on combined clinical and pathognomonic diagnostic criteria of TA evaluated using vascular Doppler and computed tomography angiography of the aorta. The patient refused to undergo surgery and received an optimal dose of chronic heart failure therapy, a high-dose steroid, and azathioprine. The patient experienced some improvements in clinical condition, functional outcome, and inflammatory markers at 1-year follow-up.

**Conclusions:**

Clinical criteria and various imaging modalities may be used to determine the etiology of MD complicating LSVA in silent TA. As an alternative to surgery, the optimal medical treatment might result in a satisfactory outcome.

## Background

Sinus of Valsalva aneurysm (SVA) is a rare condition that may appear as an acquired or congenital disease. The prevalence of SVA ranges from 0.14 to 1.5% [1], and a left SVA (LSVA) is the most infrequent [2]. Approximately 65–85% of SVAs are from the right coronary sinus, 10–30% are from the noncoronary sinus, and only < 5% are from the left coronary sinus [2]. Acquired SVAs are more infrequent than congenital ones. SVA is four times more frequent in men than in women, and the incidence of this pathology is the highest among Asians [3].

Myocardial dissection (MD) is a rare complication of an SVA. One of the acquired etiologies of SVA is Takayasu arteritis (TA), an inflammation affecting great vessels, primarily the aorta. It often remains underdiagnosed because of the silent nature of the illness. Here, we report an approach to determine the etiology of MD complicating SVA in a stable TA and its treatment.

## Case presentation

**Fig. 1:**
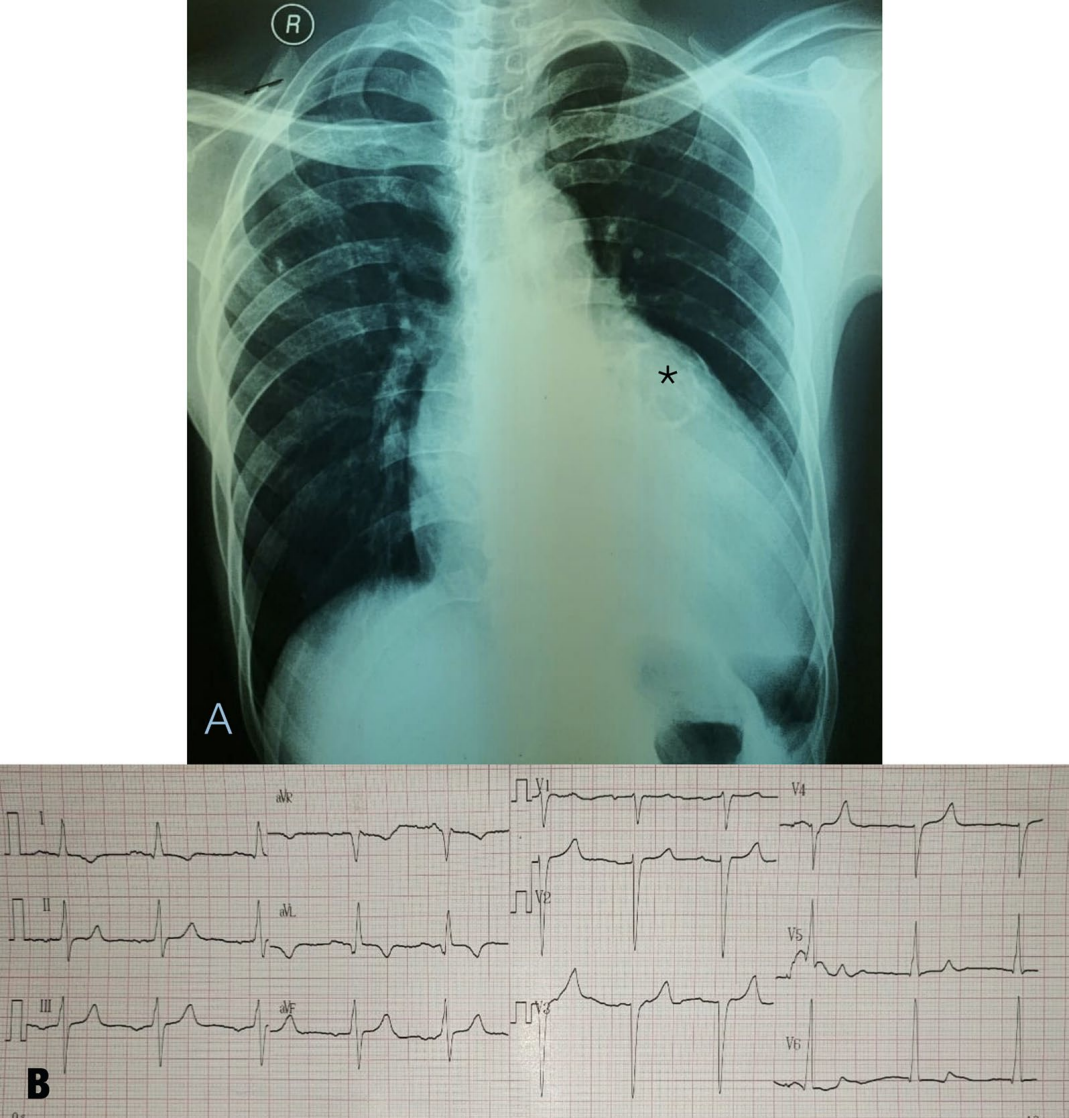
Chest X-ray and electrocardiography (ECG). Thorax posteroanterior X-ray showed cardiomegaly and circular calcification annotated with asterisks (**a**), and ECG showed poor R wave progression with left ventricular hypertrophy (**b**)

We report the case of a 41-year-old male who presented to our outpatient department with minor symptoms, including dyspnea during heavy activities. The patient did not have a history of fever, weight loss, malaise, or carotid tenderness but had a 1-year-old history of dyspnea on exertion. The patient never had any previous indications of autoimmune diseases or infections. Approximately 17 years earlier, at the age of 24, the patient experienced a frequent loss of consciousness during exercise. He had no history of hypertension, diabetes, smoking, dyslipidemia, or obesity. The patient was diagnosed with dilated cardiomyopathy a year ago, and echocardiography showed that all chambers were dilated with a left ventricular ejection fraction (LVEF) of 39%. The patient had received an angiotensin-converting enzyme inhibitor, a beta-blocker, and spironolactone and had good compliance and functional outcomes as the patient has presently been classified as New York Heart Association functional class I. Physical examinations revealed blood pressure of < 120/80 mmHg and a heart rate of 60 beats per minute (bpm) because of the optimal medical treatment he had received since last year. The patient had left carotid bruit and an enlarged heart with a grade IV/VI holosystolic murmur on the apex radiating to the axilla. Chest X-ray showed cardiomegaly, and electrocardiography revealed sinus rhythm with a heart rate of 64 bpm, poor R wave progression, and left ventricular hypertrophy (LVH) (Fig. 1). Transthoracic echocardiography (TTE) and transesophageal echocardiography (TEE) showed eccentric LVH with reduced ejection fraction and some hypoechoic lesions resembling cysts at the anterior, anteroseptal, and anterolateral walls, suggestive of myocardial detachment. Both ventricles were dilated with reduced LVEF (31%). A severe functional mitral regurgitation secondary to increased left ventricular end diastolic volume and mild aortic regurgitation due to aortic root dilatation and uncoaptation of the left coronary cusps were detected. Moreover, TTE and TEE data obtained last year showed similar results. The patient underwent cardiac computed tomography (CT), which revealed a large calcified LSVA protruding into the left ventricular anteroseptal, anterior, and anterolateral walls, causing MD. The patient had no plaque, stenosis, or intimal wall thickness on the coronary arteries. CT angiography (CTA) showed diffuse calcification from the aortic arch and main branches of the aorta, extending to the descending thoracic and abdominal aortae. The patient had mural thickness at the left carotid artery as a sign of active disease, and no calcification or thickness was observed on either renal artery on CTA. We performed duplex ultrasonography to assess the involvement of aortic branch arteries. A long-diffuse-homogenous-concentric type IV plaque was detected along the left common carotid artery without the participation of other aortic branch arteries. The venereal disease research laboratory test, treponema pallidum hemagglutination assay, anti-hepatitis B surface antigen, anti-human immunodeficiency virus (anti-HIV), and anti-streptolysin titer O were nonreactive; inflammatory markers were slightly increased, including the C-reactive protein (CRP) (0.51 mg/dL) and erythrocyte sedimentation rate (ESR) (25 mm/h).

The patient was diagnosed with TA with MD complicating LSVA and dilated cardiomyopathy and received the treatment for chronic heart failure treatment: a beta-blocker, an angiotensin-converting enzyme inhibitor, and a mineralocorticoid antagonist, which were administered at the optimal dose. The patient has also received a high-dose steroid (40-mg methylprednisolone) as the initial therapy for TA and aspirin (81 mg, daily). Later, azathioprine (50 mg, b.i.d.) was added to further reduce inflammation. The symptoms and signs were improved, and inflammatory markers decreased after 3 months of therapy. Follow-up ESR was 17 mm/h, and the CRP level decreased to 0.3 mg/dL. The patient experienced no adverse events owing to the medications.

## Discussion and conclusions

We present a rare case of LSVA causing MD to the left ventricular septal and free walls. LSVA can be broadened and cause various complications, such as coronary obstruction, MD, rupture, aortic valve incompetence, and dysrhythmia. It usually induces acute angina, dyspnea, or syncope during the onset of MD. However, the patient ensured that he never experienced such symptoms and revealed chronic heart failure symptoms only. The dissection mechanisms were postulated by myocardial ischemia or rupture due to coronary artery compression because of the aneurysm. [1] Other potential triggers of MD are infective endocarditis, myocardial infarction, and congenital anomaly of the aortic annulus.

Determining the etiology of SVA can be challenging because of various possibilities ranging from congenital to acquired conditions. Marfan syndrome is a frequently observed congenital etiology, whereas infections such as syphilis, streptococcal, HIV, and mycobacterial infections are the possible causes of the acquired version of the disease. Autoimmune diseases such as TA, systemic lupus erythematosus (SLE), and rheumatoid arthritis (RA) may also induce SVA. Moreover, it can be triggered by atherosclerotic progression or injury [2].

In our patient, Marfan syndrome was less likely to be the diagnosis; it was excluded based on the Marfan systemic score. The patient had no history of infection and revealed no signs and symptoms of streptococcal, syphilis, HIV, and mycobacterial infections. The possibility of a connective tissue disease was also eliminated as no clinical signs and symptoms of SLE and RA, among others, were observed.

**Fig. 2:**
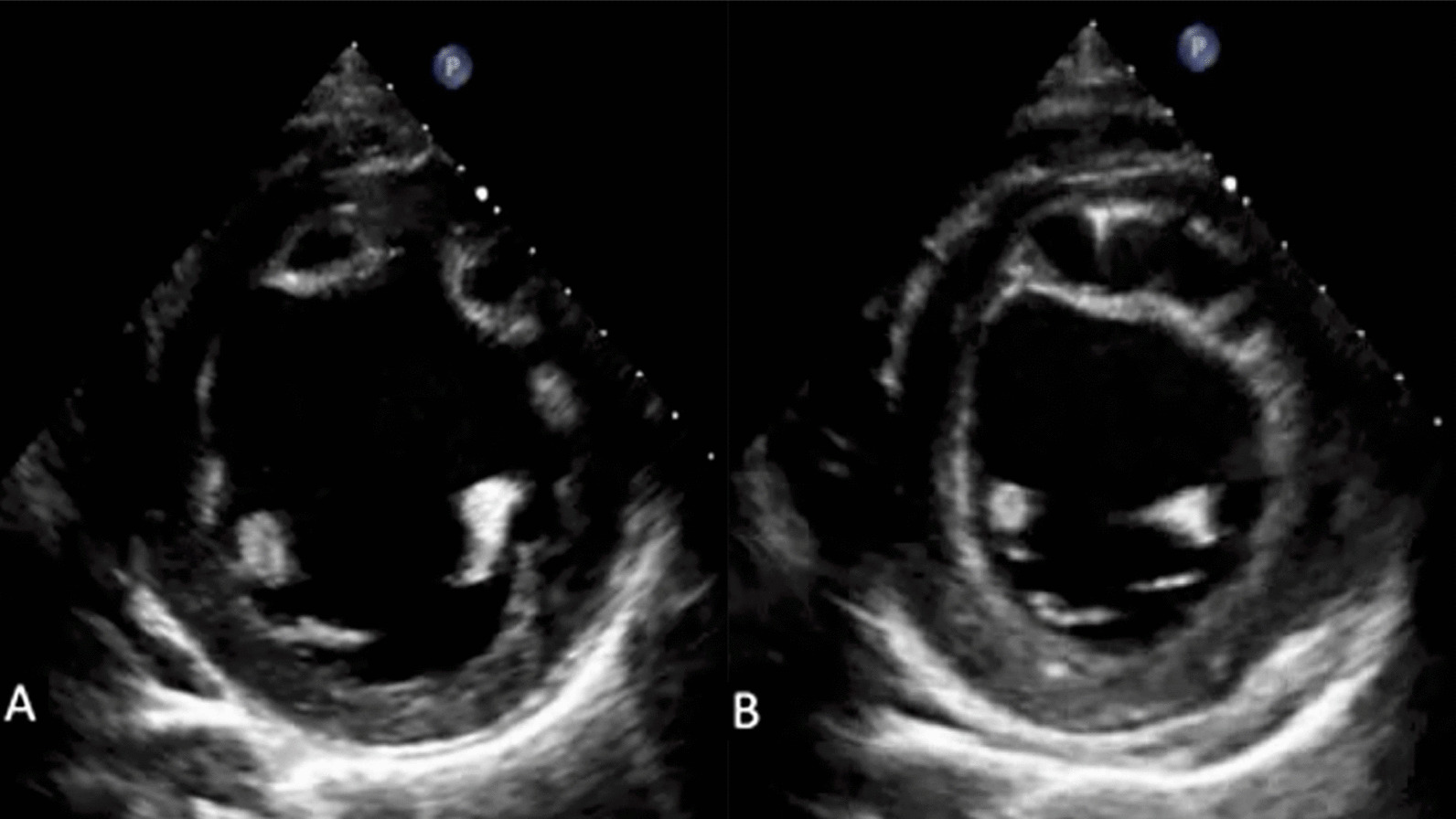
Transthoracic echocardiography. Echocardiography showed a cyst-like mass at the left anterior descending artery territory wall. The mass changed in size during diastole (**a**) and systole (**b**)

Echocardiography, either TTE or TEE, can be used to diagnose MD as it is the most accessible examination. The hollow structure in the left ventricular anterolateral wall (Fig. 2) can be differentially diagnosed with cysts or other masses. However, the structure varied in size depending on the cardiac cycle, and the color Doppler signal was filled in the cavity, which characterizes MD. Cysts or masses present in manners contradicting all features [4]. The dilated cardiomyopathy was diagnosed on echocardiography, which showed that all chambers were dilated with eccentric LVH. This might be due to volume overload in inflammatory myocarditis and disease involvement or myocardial ischemia due to the left anterior descending (LAD) artery compression. The patient had normal coronary arteries, no history of hypertension, and no stenosis at renal arteries. Severe functional mitral regurgitation occurred secondary to the increase in left ventricular end diastolic volume [5]. Aortic regurgitation quantification might be underestimated because of the involvement of multiple valves.

**Fig. 3:**
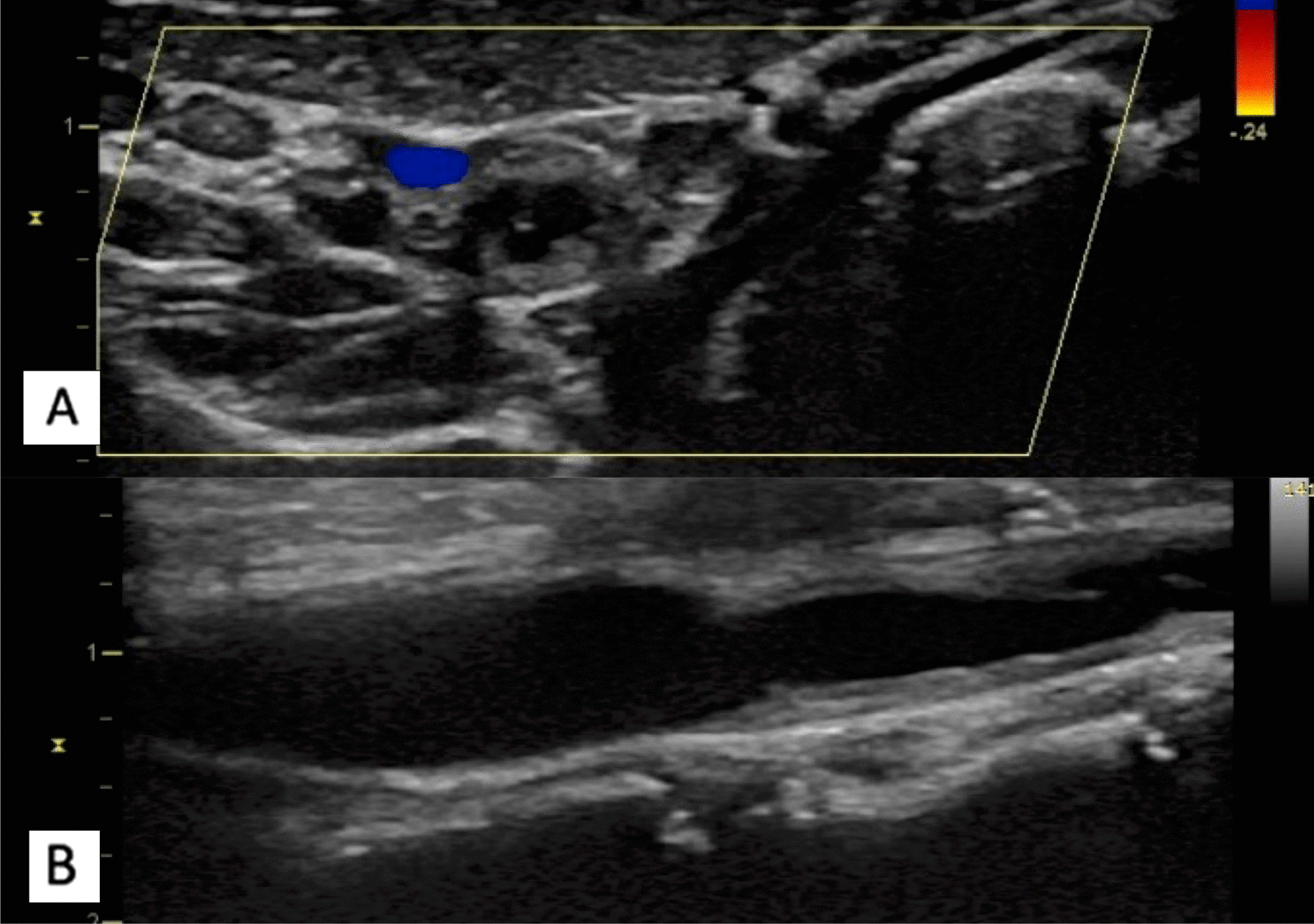
Carotid Doppler ultrasonography. Carotid ultrasonography showed a typical noncalcified homogenous lesion of Takayasu arteritis, causing an irregular vessel wall in the perpendicular (**a**) and longitudinal (**b**) views

The patient was diagnosed with TA, although the clinical manifestations were atypical. The patient met merely two of the six diagnostic criteria proposed by the American College of Rheumatology in 1990: the disease onset was before the patient reached the age of 40 years and bruit was heard around the left carotid artery [6]. At least three criteria should be met for the clinical diagnosis of TA. The patient met only one obligation criterion proposed by Ishikawa: the disease onset was before the age of 40 years; he met three minor criteria: left mid common carotid, descending thoracal and abdominal aorta lesions. Two major criteria, one major and two minor, or at least four minor criteria should be met for a confirmed diagnosis. The patient had none of the major Ishikawa criteria. However, we noted a pathognomonic characteristic of TA using duplex carotid ultrasonography and CTA. Duplex carotid ultrasonography identified a typical vascular lesion for TA in contrast with that observed in atherosclerotic plaques, which appeared nonhomogeneous and often calcified and were associated with an irregular vessel wall (Fig. 3) [7].

**Fig. 4:**
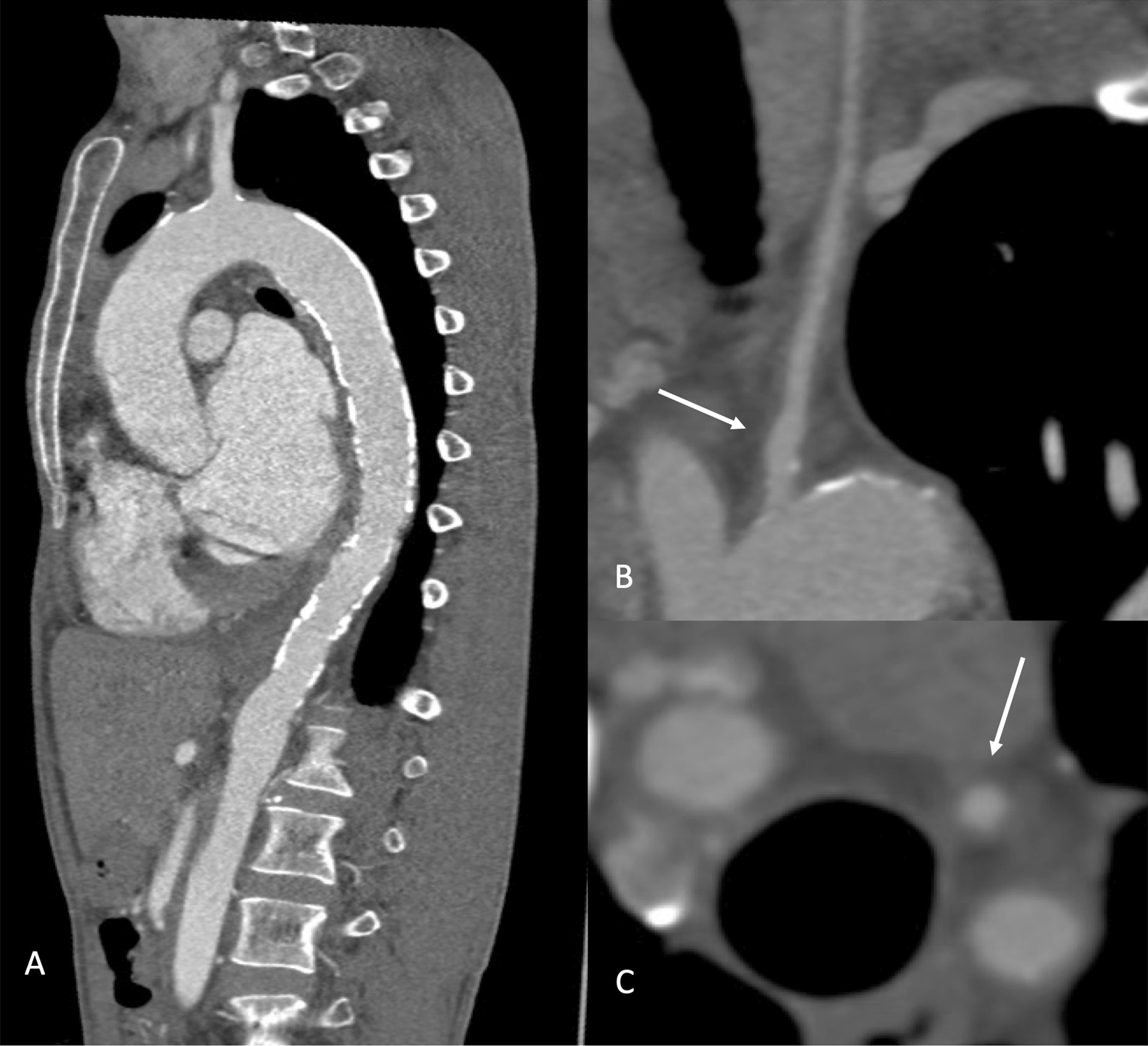
Computed tomography angiography of the aorta. Mural calcification extended from the arcus toward the abdominal aorta, characterizing the pathognomonic sign of Takayasu arteritis (TA) (**a**). An active lesion of TA was spotted in the left common carotid artery. The arrows pointed to a noncalcified mural thickening in longitudinal (**b**) and perpendicular (**c**) views

**Fig. 5:**
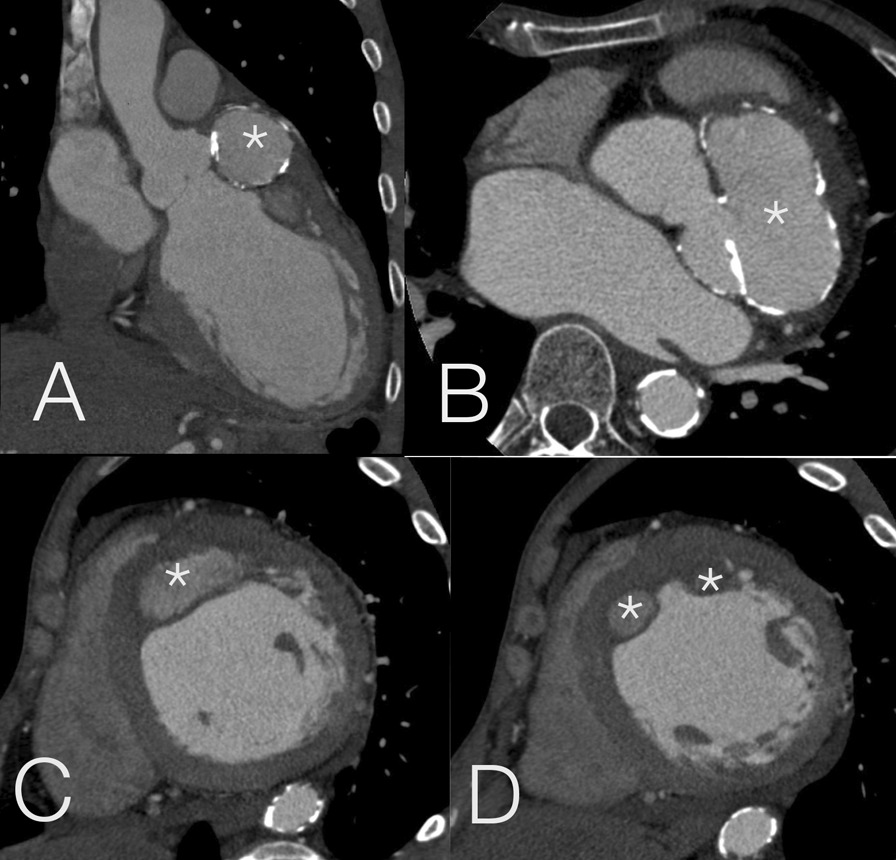
Cardiac computed tomography (CT). Left sinus of Valsalva aneurysm (LSVA) visualized using cardiac CT coronal (**a**) and axial (**b**) views, pointed by asterisks. The calcified wall of LSVA protruded and extended, forming myocardial dissection at the basal (**c**) to mid (**d**) anterior and anteroseptal walls, annotated with asterisks

CTA demonstrated diffuse mural calcification at the aortic arch and osteal of the main branches of the aorta, extending to the thoracic and infrarenal abdominal aortae, which characterize the TA pattern (Fig. 4) [8]. Diffuse mural thickening appeared on the left common carotid artery, which suggested that the lesions were still active [9]. The calcification and thickening of the mural resulted in the narrowing of three main branches of the aorta, particularly the left carotid artery; this explained the patient’ history of stroke almost a decade ago. CT also confirmed that the aneurysm expanded to the LAD territory walls without rupture into any chambers (Fig. 5), which is probably due to artery compression because the patient had normal coronary arteries.

Cardiac magnetic resonance (CMR) and magnetic resonance angiography can better differentiate the etiology of cardiomyopathy and illustrate the aortic vessel and disease activity status [10]. CMR can evaluate stenotic and aneurysmal lesions in vessel walls (i.e., thickening, edema, and degeneration) [11]. Unfortunately, our center did not have this facility; hence, we could not better evaluate the myocardium and the aorta.

TA without any fatal complications is generally associated with a good prognosis. However, MD can increase the risk of morbidity and mortality. The primary goal of disease management is to prevent its progression and aneurysm extension. To our knowledge, there is a lack of any specific guidelines or consensus regarding myocardial detachment. Patients with similar cases of MD in SVA were treated surgically [12, 13]. Patients with SVA should receive similar treatment to those with aortic root aneurysm, as stated in international guidelines [14, 15]. However, our patient refused to undergo surgery and opted for medical treatment. He showed improvement in his symptoms, vital signs, and functional outcomes a year after optimal medical therapy.

The use of anti-inflammatory drugs is indicated for active disease to prevent permanent vascular damage, whereas inactive disease is associated with disease progression. Glucocorticoids and various immunosuppressive drugs are the first-line anti-inflammatory agents. High-dose steroid therapy should be initiated immediately for inducing remission. The European League Against Rheumatism (EULAR) recommends tapering glucocorticoid dose to a target dose of 15–20 mg/day at 2–3 months and ≤ 10 mg/day at 1 year after the disease is controlled [16]. Disease-modifying antirheumatic drugs such as methotrexate, mycophenolate mofetil, leflunomide, azathioprine, and cyclophosphamide can also be added to glucocorticoid therapy or can be switched with drugs used in this therapy as per the 2018 update of the EULAR recommendations for the management of large vessel vasculitis [16]. The patient received both steroids and azathioprine. The levels of his inflammatory markers decreased, and he experienced no long-term adverse events at 1-year follow-up.

In conclusion, we reported a rare case of MD complicating LSVA in a patient with TA. Few studies have focused on determining the etiology of MD complicating SVA. TA may have various presentations. Therefore, we should consider the possibility of diagnosis based on various imaging modalities. Although our patient did not undergo surgery, his signs and symptoms and inflammatory markers improved with optimal medical treatment.

## Data Availability

Not applicable.

## Abbreviations

CRP: C-reactive protein
CT: Computed tomography
EULAR: European League Against Rheumatism
ESR: Erythrocyte sedimentation rate
HIV: Human immunodeficiency virus
LAD: Left anterior descending
LSVA: Left sinus of Valsalva aneurysm
LVH: Left ventricular hypertrophy
MD: Myocardial dissection
RA: Rheumatoid arthritis
SLE: Systemic lupus erythematosus
SVA: Sinus of Valsalva aneurysm
TA: Takayasu arteritis
TEE: Transesophageal echocardiography
TTE: Transthoracic echocardiography
